# Toward Clean and Economic Production of Highly Efficient Perovskite Solar Module Using a Cost-Effective and Low Toxic Aqueous Lead-Nitrate Precursor

**DOI:** 10.3390/nano12213783

**Published:** 2022-10-27

**Authors:** Yi-Chen Teng, Tzu-Sen Su, Shiang Lan, Ahmed Fouad Musa, Tzu-Chien Wei

**Affiliations:** 1 Department of Chemical Engineering, National Tsing Hua University, 101, Section 2, Kuang Fu Road, Hsinchu 30013, Taiwan; 2 Taiwan Perovskite Technology Co., Ltd., 2F, No. 33, Section 1, Jiafeng 11th Road, Zhubei City 302052, Taiwan

**Keywords:** perovskite solar cell, lead nitrate, module

## Abstract

Toxic substance usage remains one of the major concerns that must be addressed toward the commercialization of perovskite photovoltaics. Herein, we report a highly efficient perovskite solar module (>13%) fabricated via a wet process that uses a unique aqueous Pb(NO_3_)_2_ precursor, eliminating the use of toxic organic solvents during perovskite film preparation. In addition, we demonstrate a unique pattern in a monolithically interconnected module structure to check the uniformity of perovskite film and the quality of laser scribing. Finally, we highlight that this aqueous Pb(NO_3_)_2_ precursor protocol could achieve an enormous cost reduction over conventional PbI_2_ organic solutions whether in the laboratory research stage or at mass production scale, strengthening the core competitiveness of perovskite solar cells in the Darwinian ocean of photovoltaic technologies.

## 1. Introduction

In the last decade, the perovskite solar cell (PSC) based on organometallic halide perovskite absorbers has been studied extensively and a certificated power conversion efficiency (PCE) of 25.7% has been achieved [1], making PSC a promising technology for next-generation photovoltaics. Nevertheless, PSC technology faces considerable challenges in practical use. Firstly, the active area of high-efficiency research cells is less than 1 cm^2^. Hence, fabrication technology for high quality and large area perovskite film is urgently required. Secondly, the toxic materials, including the inorganic lead in perovskite film, and the massive amount of toxic processing solvents present tremendous environmental concerns. Finally, the cost breakdown analysis shows that the raw materials in current research cells are too expensive to be used at scale.

Aiming to resolve these challenges, the progression of the perovskite solar module (PSM) has become a hot topic recently. In particular, PSM with a monolithically connected structure is commonly chosen because it enables high voltage output and low resistive loss. In 2014, the first PSM with a PCE of 5.1% on a 16.8 cm^2^ active area was reported by Aldo Di Carlo et al. [2]. They found the poor uniformity of perovskite film prepared from spin-coating accounts for the efficiency loss when compared with small area cells (9.1% on 0.1 cm^2^ area). To mitigate this, they turned to blade coating and the PCE of a 10 cm^2^ PSM was improved to 10.3% [3]. Later on, Sang Hyuk Im et al. reported the first inverse-structured PSM with 5.1% PCE on a 40 cm^2^ active area [4]. In the same year, Thomas M. Brown et al. demonstrated a flexible PSM with 3.1% PCE on a 7.9 cm^2^ plastic substrate. Based on these pioneering works, in attempts at the implantation of new materials, new treatments and new processes that originated from small cells into PSM platforms were actively carried out. For example, Subodh G. Mhaisalkar et al. highlighted material cost issues and used ZrO_2_ and carbon to replace spiro-OMeTAD HTM and Au electrode, respectively in PSM [5]. Ruihao Chen et al. realized the concept of passivation on a CsPbI_3_-based PSM and demonstrated improved module stability [6]. Junwen Zhang et al. implemented compositional engineering of perovskite absorber by fabricating an FA-based PSM using two-step sequential blade-coating [7]. Recently, Hsin-Hsiang Huang et al. pointed out that the rapid formation of perovskite in one-step deposition can result in quality inconsistency in large-area coating and therefore they introduced sulfolane as an additive in the precursor solution to retard the formation kinetics of perovskite, providing a greater operation window for large area coating at the production scale [8]. Table 1 presents selected PSM papers published in recent years. As can be seen, solution-based wet coatings, such as spin-coating [8,9,10,11], spray-pyrolysis [12,13], blade coating [14,15,16], and slot die coating [17,18], are the dominant processes to fabricate perovskite film, although dry processes, e.g., thermal evaporation, are also developed [19,20]. In addition, the major solvent used in solution processes remains dimethylformamide (DMF), which is a carcinogenic chemical and is almost banned with regard to wide-scale use in developed countries. Considering PSM being produced in square-meter size or even larger in the future, reducing or removing DMF usage is imperative. A few studies have addressed this issue, e.g., using gamma butyrolactone (GBL) mixed with dimethyl sulfoxide (DMSO) to avoid the use of DMF [21]. However, GBL itself is an unsafe and illegal chemical that could affect nerve pathways in the brain and is therefore also troublesome in terms of large-scale use. In addition, DMSO generally seems to be an environmentally friendly solvent and is widely used in industry, but it was also found to have toxic effects when used in 1% or higher concentrations (*v*/*v*) [22]. Compared with above-mentioned DMF alternatives, water is undoubtedly a solvent without much less environmental or biological concern. However, the fabrication of PSM via a water-based process is yet to be reported.

We have developed a low toxicity protocol to fabricate PSC using aqueous lead nitrate (Pb(NO_3_)_2_) solution. By investigating the insight of the transformation mechanism from Pb(NO_3_)_2_ to MAPbI_3_, a power conversion efficiency (PCE) of 18.7% was achieved on a small cell (0.11 cm^2^) [23,24,25]. The solvents involved in fabricating perovskite film are only water and isopropanol, enabling a green fabrication process to produce PSC. Moreover, because Pb(NO_3_)_2_ is one of the starting materials to produce PbI_2_ in industry, Pb(NO_3_)_2_ thus possesses the inherent advantage of economy in production. Herein, we realize a high-efficiency PSM based on this low toxicity protocol in this work. Specifically, by designing laser scribe patterns, adjusting perovskite film thickness and material cost analysis, a PSM with 13.1% PCE (active area = 4.2 cm**^2^**) made by an environmentally friendly and economic process is achieved.

**Table 1:** Selected PSM papers and their comparison.

Year	Module Area (cm^2^)	FF	Module Efficiency (%)	Architecture	Process	Deposition Method	Perovskite Solvent	Laser Wavelength (nm)/Type	Ref.
2021	36.6	0.710	16.06	Inverted	1-step	SC + DC	DMF + DMSO	532	[8]
2021	2.2	0.699	14.57	Inverted	1-step	SDC	2-ME + DMSO	532	[17]
2020	10.6	0.684	13.03	Inverted	1-step	BC	GBL + DMSO	CO_2_ laser	[14]
2019	47.0	0.650	14.70	Regular	2-step	BC	DMF/IPA	1064	[15]
2018	63.7	0.762	16.90	Inverted	1-step	BC	DMF	532	[16]
2018	10.1	0.699	13.03	Regular	2-step	SC + DC	DMF/IPA	-	[10]
2018	25.2	0.710	14.19	Regular	2-step	SC	DMF/IPA	-	[11]
2018	31.7	0.7445	13.98	Regular	2-step	TE + SC	IPA	-	[26]
2018	149.5	0.706	11.80	Regular	1-step	SDC	DMF	-	[18]
2017	36.1	0.715	12.10	Regular	1-step	PPM	DMF	532	[27]
2017	15.3	-	8.82	Regular	1-step	SC	DMF + DMSO	1064/532	[9]
2016	3.8	0.700	11.70	Regular	1-step	USP	DMF	Mechanically scribed	[12]
2016	40.0	0.702	15.50	Regular	1-step	SP	DMF + GBL	-	[13]
	Deposited technology: Spray-pyrolysis (SP)/Ultrasonic Spray-pyrolysis (USP)/Thermally evaporation (TE)/Spin-coating (SC)/Dip-coating (DC)/Blade coating (BC)/Pressure processing method (PPM)/Slot-die coating (SDC) Solvent: Iso-propanaol (IPA)/Dimethylformamide (DMF)/Gammabuthyrolactone (GBL)/Dimethyl sulfoxide (DMSO)/2-methoxy-ethanol (2-ME)								

## 2. Experimental Section

### 2.1. Perovskite Module Fabrication

A P1 scribing pattern of 1 mm width on the 3 × 3 cm fluoride-doped tin oxide (FTO, 2.2 mm, 8 Ω/sq, Dyesol, Queanbeyan, Australia) glass sheets was patterned by laser etching (Fiber layer, maximum laser fluence = 2.5 J/cm^2^, wavelength = 1064 nm, laser beam diameter = 20 μm, MSB-20 F, Laser Life Company, Hsinchu, Taiwan). Then, patterned substrates were ultrasonically cleaned with 2% detergent solution (PK-LCG46, Parker Co., Inc., New Taipei City, Taiwan) diluted in water and then cleaned in deionized water for 30 min in sequence. Cleaned FTO substrates were exposed to a UV–Ozone environment for 15 min before use. A TiO_2_ compact layer was deposited on a cleaned FTO substrate by spin-coating using a solution composed of 0.3 M titanium diisopropoxide bis(acetylacetonate) (75% in IPA, Sigma-Aldrich, Burlington, MA, USA) diluted in 2-propanol (IPA, 99.5%, Sigma-Aldrich, Burlington, MA, USA) at 4000 rpm for 30 s. The coated FTO substrate was then dried on a 120 °C hotplate for 5 min, followed by calcination at 500 °C for 30 min. After slowly cooling to ambient temperature, the compact TiO_2_ coated substrates were treated with a titanium tetrachloride (TiCl_4_, 99.9%, Showa, Taichung City, Taiwan) chemical bath deposition. Substrates were immersed in a 40 mM TiCl_4_ solution at 70 °C for 30 min, followed by mild rinsing with water and ethanol in sequence. After drying in the air, a TiO_2_ paste (particle size of 30 nm, Greatcell, Queanbeyan, Australia) diluted in ethanol (TiO_2_ paste/ethanol = 1/7, *w*/*w*) was spin-coated on the compact TiO_2_-coated FTO substrate at 6000 rpm for 30 s to form a mesoporous TiO_2_ film with a thickness of 150–200 nm. After coating, the bilayer film was dried at 120 °C for 5 min and then sintered again at 500 °C for 30 min.

For Pb(NO_3_)_2_/water-based PSMs, the as-prepared FTO/bilayer TiO_2_ film was treated under an UV-ozone exposure for 20 min. Subsequently, an aqueous solution containing different concentrations of 1.5 and 1.6 M Pb(NO_3_)_2_ (99%, Alfa Aesar, Ward Hill, MA, USA) was prepared and subject to ultrasonic treatment for 10 min before spin-coating onto the substrate at 6000 rpm for 10 s in a dynamic way, then dried on a hot plate at 70 °C for 30 min. After cooling to room temperature, the Pb(NO_3_)_2_-infiltrated mesoporous TiO_2_ film was immersed in an IPA solution containing 101.3 mM MAX, X = I or Cl (the molar ratio of MAI/MACl is 4.25/1), for 500, 600, and 700 s. The first dipping period was 200 s and then 100 s for each period to finish all dipping periods, which was disclosed in our previous study [25]. In each dipping cycle, the substrate was dipped into MAX solution for the desired period, rinsed by IPA solution, and then dried by high-speed spinning (6000 rpm for 10 s). Finally, the as-prepared perovskite film was annealed at 120 °C for 20 min on a hot plate.

For PbI_2_/DMF-based PSCs, PbI_2_ (99%, Sigma-Aldrich) was dissolved in a mixed solvent composed of DMF (99.8%, J.T Backer, Radnor Township, PA, USA) and DMSO (>99.6%, Sigma-Aldrich) in a volume ratio of 9/1 to form a 1.5 M PbI_2_ solution. The PbI_2_ solution was preheated at 70 °C and maintained at the same temperature throughout the whole coating process to ensure good solubility. The PbI_2_ solution was deposited on the as-prepared FTO/bilayer TiO_2_ film by spin-coating at 6000 rpm for 10 s in a static way and then dried at 70 °C for 10 min. After cooling, the film was immersed in a 70.1 mM MAX, X = I or Cl (the molar ratio of MAI (99%, Lumtec, Hsinchu, Taiwan)/MACl (99%, Lumtec) is 4.23/1), for 2 min. Finally, the perovskite film was dried and annealed at 120 °C for 20 min.

After the as-prepared FTO/bilayer TiO_2_/perovskite film cooled to room temperature, a layer of hole transport material (HTM) was spin-coated on the top at a condition of 4000 rpm for 25 s. The HTM solution was composed of 75 mM 2,2’,7,7’-Tetrakis-(N,N-di-4-methoxyphenylamino)-9,9’-spirobifluorene (Spiro-OMeTAD, 99.5%, Lumtec, Hsinchu, Taiwan), 32 mM lithium bis(trifluoromethylsulphonyl)imide (Li-TFSI, >99%, Sigma-Aldrich), and 195 mM tert-butylpyridine (tBP, >96%, Sigma-Aldrich) in chlorobenzene (99.8%, Sigma-Aldrich). P2 laser scribing has been operated to remove the bilayer TiO_2_/perovskite/HTM layers with laser frequency = 20 kHz, laser fluence = 0.4375 J/cm^2^, focal length = 89–90 mm, and scan speed = 150 mm/s. A gold back electrode was deposited on top of the Spiro-OMeTAD layer by thermal evaporation to a thickness of 80 nm. Finally, P3 laser scribing was undertaken with the aim to separate the gold back electrode.

### 2.2. Characterization and Measurement

The prepared PSCs and PSMs were measured using a 300 W Xenon light source from Peccell (PEC-L15, Peccell Technologies, Yokohama, Japan). The spectral mismatch between AM 1.5G (1000 W/m^2^) and the solar simulator was calibrated by a monocrystalline silicon photodiode (Oriel, Los Angeles, CA, USA). The Keithley 2400 was used for the current-voltage scan by applying an external voltage bias and measuring the response current with a scan rate of 10 mV/s. The cells were masked with a black metal mask with an area of 0.1 cm^2^ for a small device and 1.4 cm^2^ for a single cell in a module. The active area was 0.1 cm^2^ for a small device and 1.4 cm^2^ for a single cell in a module. No preconditioning (e.g., bias and light soaking) was used for the photovoltaic measurement. 

The morphologies of perovskite films were characterized using a high-resolution scanning electron microscope (FlexSEM 1000, HITACHI, Ibaraki, Japan) with an in-lens detector. For XRD measurement, the diffraction pattern was measured by a Rigaku Ultima IV X-ray diffractometer equipped with a ceramic tube (Cu, Kα, λ = 1.5418 Å) and an optional D/teX Ultra-high-speed, position-sensitive detector system.

## 3. Results and Discussion

### 3.1. Laser Scribing (P1-P2-P3) Adjustment

**Figure 1:**
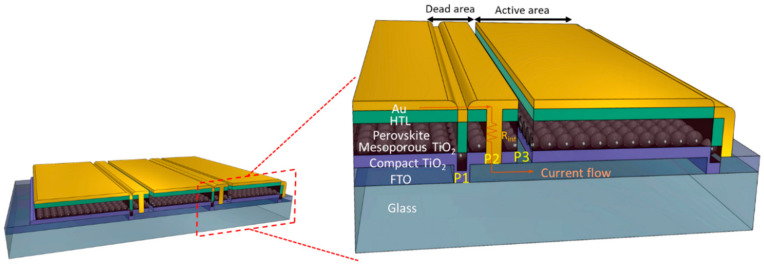
The schematics of the cross-section of the serial interconnect region (P1-P2-P3) of a PSM.

To increase the open-circuit potential (V_OC_), monolithic interconnection, also known as P1-P2-P3 process, is commonly used in PSM. The P1-P2-P3 process consists of three scribing processes to form the interconnection between cells [28,29]. Figure 1 illustrates a typical monolithic PSM in which the interconnection region is highlighted. Accordingly, the P1 scribing insulates the conductive substrate of neighboring cells. The P2 process forms an interconnection between the front contact and the gold electrode. The P3 process removes the front contact from the gold electrode to the FTO substrate to physically isolate the neighboring cells, completing a series of connections. The entire P1-P2-P3 region is photovoltaic inactive, so optimizing this “dead” area is crucial to maximizing the power output when upscaling a PSM [30]. Meanwhile, the quality of P1, P2, and P3 scribing determines the electrical insulation of a PSM, thus influencing the overall performance significantly. It has been reported that laser wavelength and laser pulse are two key factors for effective scribing. According to the absorbing characteristics of the perovskite absorber, most of the energy generated by 355 nm and 532 nm will be absorbed by the perovskite layer, avoiding damage to the conductive substrate. The 355 nm and 532 nm lasers featuring nanosecond or picosecond pulse are more suitable than the 1064 nm one for scribing the perovskite layer, although the 355 nm or 532 nm laser machine is generally more expensive [31]. Furthermore, the 1064 nm laser is reported to damage the conductive coating of the substrate more easily [32]. Laser scribing technology is known to remove the substance on the substrate in two patterns, the laser-irradiated zone (LIZ) and the laser-affected zone (LAZ). The width of LIZ is approximately equal to the beam width of the laser, but the width of LAZ can be associated with the pulse width of the laser beam and the heat dissipation capability of the substrate. Since the total scribing width (L) is the algebraic summation of the width of LIZ and LAZ, it is usually imperative to use a high-cost laser source with nanosecond or even picosecond pulse width to minimize the area of LAZ [33,34]. In Table 1, because of the damage concern on the conductive substrate, the 532 nm wavelength of laser for P2 scribing is most often used. To our knowledge, there are few papers using consumer-grade 1064 nm laser to prepare the P1-P2-P3 process in PSM and further investigating the effects in detail. Herein, we realize the use of a cost-effective laboratory fiber laser with a wavelength of 1064 nm and mere 50 μs pulse width to pattern P1-P2-P3 with a satisfactory performance by turning the focal length, power level, and scanning speed, demonstrating the capability to study PSM under a limited budget.

**Figure 2:**
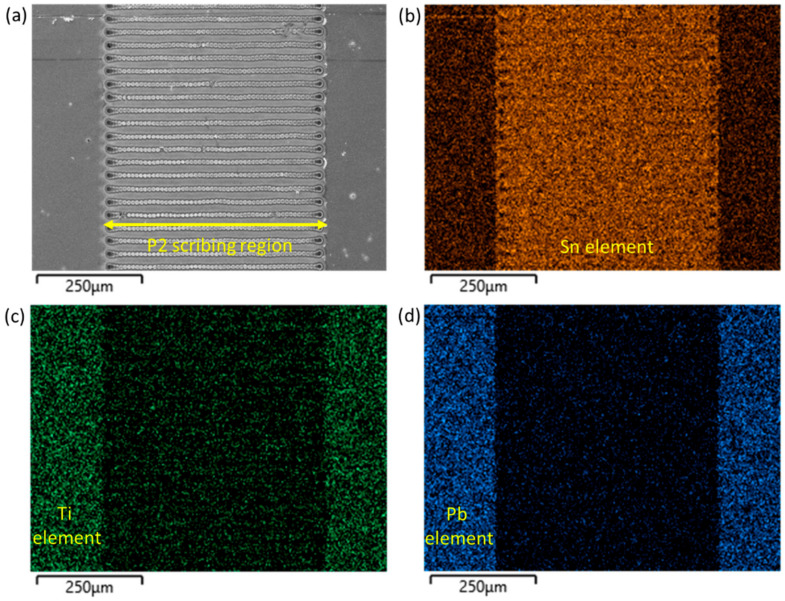
(**a**) SEM image and corresponding elemental mapping (**b**) Sn signal (**c**) Ti signal (**d**) Pb signal of P2 scribing.

**Figure 3:**
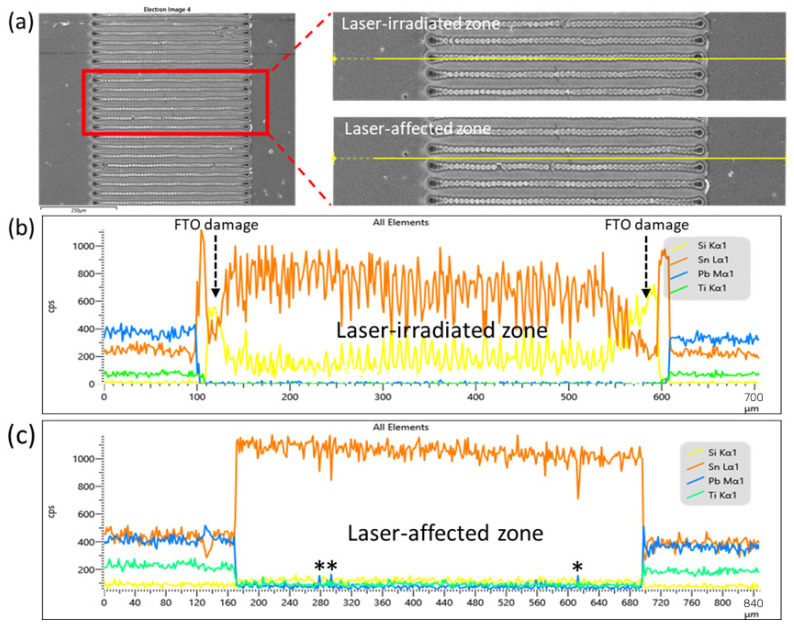
(**a**) SEM images of P2 scribing and EDX line scans on (**b**) LIZ and (**c**) LAZ. PSM architecture for individual cell measurement. Note: * is marked occasional Pb peak signal in LAZ.

The pulse width is directly relative to the influence of thermal processes in perovskite removal [35]. It has been reported that the material ablation by laser will generate the electron-phonon and then transfer to heat. The pulse width lower than 100 ps could avoid the heat conduction to the surrounding area and decrease the damage [36]. Because the pulse width of the laser source used in this study is only 50 μs, the LAZ could occupy even more space than LIZ [37], leading to a wide LAZ and more importantly poor depth control of the whole P2 zone. To ensure insulation and minimize the impact of LAZ, the scribing pathway is purposely designed to be perpendicular instead of parallel to P2 line (shown in Figure 2). Ideally, gold electrodes would directly contact with FTO after P2 scribing. However, due to possible damage of FTO and possible residues of HTM/perovskite/ETM, a non-negligible resistance, R_int_, could exist on the surface as shown highlighted in Figure 1. R_int_ is part of overall series resistance and should be minimized. Control of R_int_ can be done by tuning the focal length at the *Z* axis (Figure S1), followed by elemental mapping in SEM observation. As shown in Figure 2, the optimal focal length can be determined by examining the continuity of Sn signal (orange, representing FTO layer), with the disappearance of Ti (green, representing TiO_2_ ETM) and Pb (blue, representing perovskite layer) in EDX mapping of the sample. It is also found when the focal length slightly increased by 1 mm (from 89 mm to 90 mm, in this case), FTO would be damaged because there are some dark dots with significant contrast in Figure S2. To further understand the quality of P2 line, EDX line scans on LIZ and LAZ were examined. Figure 3 is a zoom-in image of a P2 zone, on which EDX line scans on randomly picked LIZ and LAZ are shown. It can be found in Figure 3b that, although Sn is the major signal in the entire LIZ with lower intensity than LAZ, Si signal is also detected in it, indicating that FTO has been damaged. By contrast, no Si signal is detected in LAZ and the Sn signal is also stronger than in LIZ (Figure 3c), showing that FTO is undamaged. However, Pb is occasionally detected and marked with an asterisk in LAZ, suggesting that island-like perovskite or its decomposed residues may be not completely removed. The damage of FTO in LIZ and Pb-contains residues increase R_int_ in PSM, lowering FF. Notwithstanding this imperfection, using a cheap 1064 nm fiber laser for P2 scribing is experimentally feasible after tuning the focal length of the laser beam.

### 3.2. PSM Architecture for Individual Cell Measurement

**Figure 4:**
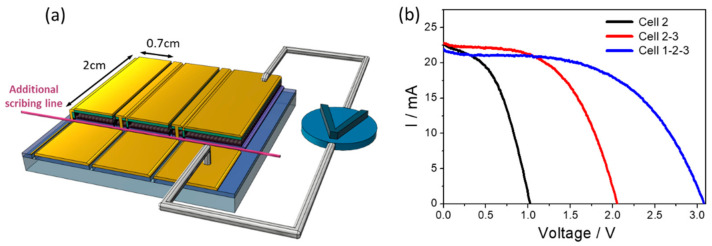
(**a**) Schematic diagram of the unique pattern for I–V measurement. (**b**) IV curves of single cell (Cell 2), two cells (Cell 2-3) and three cells (Cell 1-2-3) in series refer to the PbI_2_/DMF device.

The V_OC_ of a monolithic-structured PSM is the summation of the individual cell, but the J_SC_ of it is determined by the lowest J_SC_ within cell strips. Generally, the former is affected by P1-P2-P3 quality and the latter is related to the uniformity of perovskite film. Typical monolithic-structured PSM does not equip a contacting pad for individual cell strip measurement. Herein, a modified PSM pattern is designed to measure individual cell strip in a monolithic-structured PSM (as illustrated in Figure 4), in which an additional scribing line is applied across all cell strips. With this modification, IV measurement of individual cell strips is made possible by simply wiring the upper gold pad and bottom cell strip as terminals. In the compromise of this PSM pattern as illustrated in Figure S3, a longer resistive path on FTO when measuring a single strip causes a lower FF. In the following sections, a 4.2 cm^2^ PSM containing 3 cells with a 1.4 cm^2^ (0.7 × 2 cm^2^) active area in each was studied.

### 3.3. Effects of Problematic P2 Scribing and Inhomogeneous Spin-Coated Film

To verify the benefits of the modified PSM pattern, an exemplary PSM made from PbI_2_/DMF protocol was fabricated and tested (Figure 4b). Table 2 summarizes IV characteristics of the individual, paired and whole cells of the PSM. As can be seen, every single cell exhibits different IV parameters, in which Cell 1 and Cell 3 located on the side of the PSM express considerably lower FF than that of Cell 2, explaining the edge deforming feature of the spin-coating method. In addition, two representative features of series connection are revealed: the first one is overall V_OC_ as the algebraic summation of individual V_OC_, indicating 1064 nm laser scribing is capable to use after carefully tuning; the other is overall J_SC_ determined by the lowest J_SC_ within the individual cell (Cell 1 in this case).

Besides examining the fundamental principle of series connection, this modified PSM design also enables us to check the quality of the large-area film. Herein, an inhomogeneous perovskite film was purposely prepared by tilting the substrate during spin-coating. IV curves and IV parameters of this PSM are shown in Figure S4 and Table 3. It can be seen that J_SC_ of Cell 1 (16.3 mA/cm^2^) is considerably lower than Cell 2 and 3 (18.3 and 17.3 mA/cm^2^, respectively), rendering J_SC_ of series connecting cell 1 and 2 (Cell 1-2) and entire module (Cell 1-2-3) similarly low J_SC_ of 15.9 and 16.3 mA/cm^2^, respectively. V_OC_ drops a little but remains addable, suggesting that device integrity is maintained. The increase of FF in series connecting cell 1 and 2 (Cell 1-2) and cell 2 and 3 (Cell 2-3) is due to the shorter resistive path on FTO when measuring two strips compared to a single strip (Figure S3). In other words, the overall photovoltaic performance of this PSM is demoted only due to a single low-J_SC_ cell. Learning from this, the uniformity of perovskite film is one of the most important technical issues when upscaling the device size.

The modified PSM design can also be used to examine the impact of the problematic P2 scribing. A PSM with problematic P2 scribing between Cell 2 and Cell 3 is made and its IV performance is shown in Table 3 and Figure S5. It is found that the problematic P2 scribing significantly drops the FF but not the I_SC_ and V_OC_. The FFs of the Cell 2-3 and Cell 1-2-3 are demoted when compared with FF of Cell 1-2, while V_OC_ and J_SC_ remain addable and stable, respectively.

### 3.4. Comparison of PbI_2_/DMF and Pb(NO_3_)_2_/Water Two Step Dipping Process

**Figure 5:**
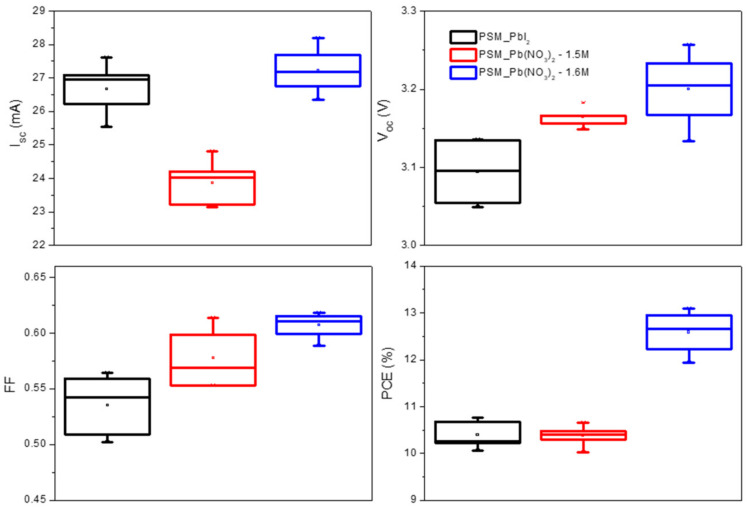
The box plot of IV measurement of PSM_PbI_2_, 1.5 M and 1.6 M PSM_Pb(NO_3_)_2_ two step dipping procedure.

**Figure 6:**
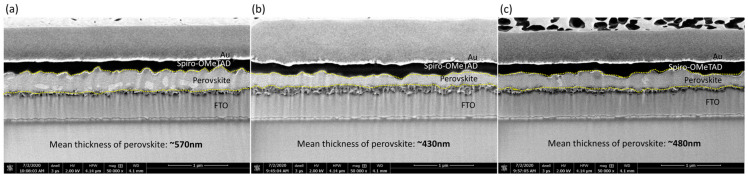
The cross-sectional SEM of full devices of (**a**) PSM_PbI_2_ and (**b**) 1.5 M (**c**) 1.6 M PSM_Pb(NO_3_)_2_ two-step dipping procedure.

PSMs made from aqueous Pb(NO_3_)_2_ protocol were fabricated [15a, 15b, 23]. Specifically, spin-coated Pb(NO_3_)_2_ film was dipped into an IPA solution containing MAX (methylammonium iodide mixed with methylammonium chloride) and rinsed in pure IPA for several cycles. The damage of FTO in LIZ and residues of Pb-contains increase R*_int_* in PSM, lowering FF for the conversion of MAPbI_3_ film. Due to the necessity of NO_3_- removal, spin-coating MAX solution onto Pb(NO_3_)_2_ film is unsuitable, although it is common in conventional PbI_2_/DMF protocol in small device fabrication. Considering the edge-deforming feature of spin-coating, PSMs made by PbI_2_/DMF protocol using a similar dipping process were also fabricated for comparison. Details of fabrication can be found in the experimental section. IV characteristics of PSMs made from Pb(NO_3_)_2_/water protocol (hereafter denoted as PSM_Pb(NO_3_)_2_) and from PbI_2_ DMF protocol (hereafter denoted as PSM_PbI_2_) are shown in Table 4 and Figure 5. As can be seen, PSM_Pb(NO_3_)_2_ exhibits higher V_OC_ and FF but lower I_SC_ than those of PSM_PbI_2_. The PCE of PSM_Pb(NO_3_)_2_ shows a champion and average PCE of 10.7% and 10.4%, respectively; both are slightly lower than PCE obtained from PSM_PbI_2_. Lower V_OC_ and FF of PSM_PbI_2_ can be attributed to its uneven perovskite film surface, which is a result of Ostwald ripening during MAX dipping. The uneven perovskite with poor contact might lead to a lower FF than the uniform film [38] and the solution method of HTM may not cover the perovskite completely, which supplies an opportunity for direct contact between perovskite and metal electrode, causing non-radiative recombination and reducing the Voc [39]. However, because the Pb(NO_3_)_2_ has to convert to PbI_2_ first and then transfer to MAPbI_3_, the reaction kinetic in Pb(NO_3_)_2_ perovskite is slower than in PbI_2_ perovskite during MAX dipping, avoiding the Ostwald ripening. Through cross-sectional SEM image analysis, higher I*_SC_* in PSM_PbI_2_ is due to its thicker MAPbI_3_ layer (~570 nm) when compared with the perovskite film thickness of PSM_Pb(NO_3_)_2_ (~430 nm) (Figure 6b). XRD analysis (Figure S6) on the perovskite film of PSM_PbI_2_ and PSM_Pb(NO_3_)_2_ reveals that PSM_PbI_2_ has a lower conversion rate of MAPbI_3_ when compared with PSM_Pb(NO_3_)_2_, which accounts for lower V_OC_ and FF of PSM_PbI_2_. Overall, the average PCE of PSM_Pb(NO_3_)_2_ is slightly lower than that of PSM_PbI_2_. It should be emphasized that even though the PSM_PbI_2_ only slightly outperforms PSM_Pb(NO_3_)_2_ under current fabrication conditions, this does not mean that the best performance of the PbI_2_/DMF system has been reached because each method can be further optimized individually. Herein, we are more interested in improving the performance of the Pb(NO_3_)_2_/water system.

Current J_SC_ of PSM_Pb(NO_3_)_2_ is less than 20 mA/cm^2^, which is ∼20% lower than Pb(NO_3_)_2_-based small cell [23,24,25,40]. The inferior J_SC_ can be mainly attributed to thin perovskite layer thickness with hills and valleys (∼430 nm for Figure 6b), which results in insufficient light harvesting. Therefore increasing the thickness of the perovskite layer becomes an urgent action to enhance the light harvesting as well as the PCE of PSM_Pb(NO_3_)_2_. The solubility of Pb(NO_3_)_2_ in water is approximately 1.8 M at 25 °C. Considering the concentration of Pb(NO_3_)_2_ solution for current PSM_Pb(NO_3_)_2_ is 1.5 M, it seems there exists ample room to use a more concentrated solution to obtain a thicker spin-coated film. The photograph of spin-coated Pb(NO_3_)_2_ films made by 1.5 M (initial condition), 1.6 M, 1.7 M, and 1.8 M Pb(NO_3_)_2_ aqueous solution are shown in Figure S7. As can be seen, Pb(NO_3_)_2_ films made from 1.7 m and 1.8 M exhibit apparent pitfalls even by the naked eyes, which are small Pb(NO_3_)_2_ crystals seeding out due to saturation during spin-coating and drying. However, the higher concentration of Pb(NO*_3_*)*_2_*/water could provide an additive to improve the film quality and this will be investigated in future studies. Consequently, 1.6 M is a safe choice. The thickness of perovskite film made by 1.6 M Pb(NO_3_)_2_ solution becomes ~480 nm (Figure 6c), which is thicker than the pristine one made by 1.5 M Pb(NO_3_)_2_ solution by approximately 50 nm, benefiting the light harvesting by the thicker layer and then improving the photocurrent. The IV characteristics of PSM_Pb(NO_3_)_2_ made from 1.6 M Pb(NO_3_)_2_ solution are compared in Table 4 and Figure 5. Benefitting from the thickened perovskite layer, all photovoltaic parameters, including V_OC_, I*_SC_*, and FF, were improved considerably. The champion PCE of 1.6 M Pb(NO_3_)_2_ based PSM achieved 13.1%, which is the highest performance of PSM using a low toxicity aqueous protocol ever reported. If we compare the performance of 1.6 M Pb(NO_3_)_2_ based PSM and J_SC_ of Pb(NO_3_)_2_ based small cell (fabricated using the same process conditions), the J_SC_ deviation decreases to reach less than 10% (20.1 versus 21.8 mA/cm^2^). The major difference between small cell and PSM is FF, which is originated from the perfection of P1-P2-P3 structure and warrants deep study and investment in more suitable hardware, such as 532 nm, picosecond laser, or automatic alignment XY table.

### 3.5. Breakdown of Materials of Pb-Precursor Film of Two-Step Fabrication

**Figure 7:**
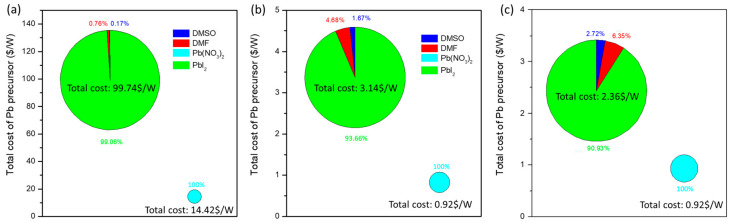
Cost analysis of lead precursor (**a**) based on our results and academic research; PCE = 10.75% for PSM_PbI_2_; PCE = 13.1% for PSM_Pb(NO_3_)_2_. (**b**) Based on industrial production PCE = 15% for both of PSM_PbI_2_ and PSM_Pb(NO_3_)_2_. (**c**) Based on industrial production PCE = 20% for PSM_PbI_2_ and PCE = 15% for PSM_Pb(NO_3_)_2_.

The cost of raw materials to fabricate perovskite film made from Pb(NO_3_)_2_/water and PbI_2_/DMF process based on current fabrication condition and the optimistic forecast is analyzed. Because both processes share identical MAX solutions, raw material costs involved in depositing Pb-contained film in the first step are compared. Prices of the chemicals are taken from the website of Sigma-Aldrich company, in which chemicals of reagent grade (high purity) were used in current fabrication and chemicals of industrial grade (less pure) were implemented for mass production. The result is shown in Figure 7, and details of the calculation are provided in Tables S1 and S2. Figure 7a is the analysis of material cost to deposit Pb-precursor film based on current result (PCE of PSM_ Pb(NO_3_)_2_ and PSM_PbI_2_ are 13.1% and 10.8%) and it shows that the material used for casting Pb(NO_3_)_2_ film is 14.4 USD/W, which is nearly seven times lower than common PbI_2_/DMF process (99.7 USD/W). From the detailed comparison shown in Table S1, the advantage is obviously obtained from the Pb-precursor price in which the reagent grade of Pb(NO_3_)_2_ is 6.0 USD/g but PbI_2_ of the same purity grade is 25.8 USD/g.

Forecasting mass production, with the assumption of identical 15% PCE of PSM using chemicals from industrial grade purity, the material cost to deposit Pb-precursor film of Pb(NO_3_)_2_/water and PbI_2_/DMF process decreases to 0.9 and 3.1 USD/W, respectively (Figure 7b and Table S2). In addition, because the fabrication of PbI_2_/DMF process is more mature than Pb(NO_3_)_2_/water process, we further compare 20% PCE of PbI_2_/DMF PSM prepared by industrial grade purity of chemicals and 15% PCE of Pb(NO_3_)_2_/water PSM. (Figure 7c and Table S3) The triumph in the economic production of the Pb(NO_3_)_2_/water process remains evidently. It is worth noting that, in the PbI_2_/DMF process, the price of DMF and DMSO accounts for >10% of the total cost (0.018/0.162), evidencing that the use of DMF/DMSO as solvent not only induces toxicity concerns, but also presents a disadvantage in terms of cost for mass production. Considering Pb(NO_3_)_2_ is usually used as the starting material to synthesize PbI_2_ via a well-known “golden rain reaction” in lead chemistry, it is obvious that using Pb(NO_3_)_2_ as the precursor for PSM is always more economic against PbI_2_. This triumph is expected to maintain effectiveness even as the deposition method advances from laboratory spin-coating to industrial slit coating.

## 4. Conclusions

We report a groundbreaking strategy for the eco-friendly and cost-effective fabrication of highly efficient PSMs. It is demonstrated that the toxic solvent used in the fabrication process of a PSM could be fully eliminated, enabling the production of PSC via green chemical engineering. Moreover, given the fact that Pb(NO_3_)_2_ has an inherently lower cost than PbI_2_, the aqueous Pb(NO_3_)_2_ precursor presents a great deal of potential to further enhance the advantage of PSC technology. The current result of PSM_Pb(NO_3_)_2_ exhibits 13.1% in PCE using a laboratory grade 1064-nm laser. Further enhancement of the Pb(NO_3_)_2_/water protocol can be easily realized in the following tasks: (1) Invest in an appropriate laser scribing machine to improve the quality of P1-P2-P3 structure. (2) Increase the thickness of perovskite film using a large-area processable filming method, such as slit coating, so that J_SC_ of PSM can be increased. (3) Undertake compositional engineering of perovskite composition by incorporating FA^+^, Cs^+^ cations, and Br^-^ anion to enhance the photovoltaic performance as well as stability of perovskite absorber.

## Figures and Tables

**Table 2:** Photovoltaic parameter of each single cell and interconnected cells of a PbI_2_-based PSM.

Cell	I_SC_ (mA)	J_SC_ (mA/cm^2^)	V_OC_ (V)	FF	P_max_ (mW)	PCE (%)	Active Area (cm^2^)
1	21.8	15.6	1.04	0.38	8.8	6.2	1.4
2	22.8	16.3	1.02	0.46	10.8	7.7	1.4
3	23.1	16.5	1.03	0.43	10.3	7.4	1.4
1-2	21.9	15.6	2.00	0.50	21.8	7.8	2.8
2-3	22.9	16.4	2.05	0.54	25.3	9.1	2.8
1-2-3	22.0	15.7	3.08	0.54	36.5	8.7	4.2

Note: The perovskite layer was made by a 2-step method from PbI_2_ (1.5 M) in anhydrous DMF:DMSO = 9:1 (*v*:*v*).

**Table 3:** Photovoltaic parameter of each single cell and interconnected cell.

Sample	Type	I_SC_ (mA)	J_SC_ (mA/cm^2^)	V_OC_ (V)	FF	PCE (%)
PSM with inhomogeneous perovskite film (~450 nm)	1	22.8	16.3	1.07	0.41	7.1
	2	25.6	18.3	1.07	0.48	9.4
	3	24.2	17.3	1.01	0.50	8.8
	1-2	22.3	15.9	2.09	0.55	9.1
	2-3	24.4	17.3	2.06	0.57	10.3
	1-2-3	22.8	16.3	3.06	0.57	9.4
PSM contains problematic P2 structure between Cell 2 and Cell 3 (~600)	1	29.6	21.1	1.01	0.31	6.6
	2	30.0	21.4	0.99	0.39	8.3
	3	29.8	21.3	0.98	0.40	8.4
	1-2	29.9	21.4	1.98	0.42	9.0
	2-3	29.1	20.8	2.03	0.24	5.1
	1-2-3	29.3	20.9	2.99	0.30	6.2

Note: The perovskite layer was made by a 2-step method from PbI_2_ (1.5 M) in anhydrous DMF:DMSO = 9:1 (*v*:*v*).

**Table 4:** Photovoltaic parameter of PSM_PbI_2_, 1.5 M and 1.6 M PSM_Pb(NO_3_)_2_ two step dipping procedure.

Type		I_SC_ (mA)	J_SC_ (mA/cm^2^)	V_OC_ (V)	FF	PCE (%)
PSM_PbI_2_	Average	26.7 ± 0.72	19.05 ± 0.72	3.10 ± 0.034	0.54 ± 0.026	10.4 ± 0.27
	Champion	25.5	18.2	3.14	0.56	10.8
1.5 M PSM_Pb(NO_3_)_2_	Average	23.9 ± 0.63	17.1 ± 0.63	3.16 ± 0.012	0.58 ± 0.025	10.4 ± 0.21
	Champion	23.1	16.5	3.16	0.61	10.7
1.6 M PSM_Pb(NO_3_)_2_	Average	27.2 ± 0.65	19.4 ± 0.65	3.20 ± 0.044	0.61 ± 0.011	12.6 ± 0.43
	Champion	28.2	20.1	3.20	0.61	13.1

## Data Availability

Not applicable.

## Supplementary Materials

The following supporting information can be downloaded at: https://www.mdpi.com/article/10.3390/nano12213783/s1, Figure S1: The control of focal length at Z axis; Figure S2: The optical microscopy images of 89 and 90 mm focal length of P2 scribing on FTO/c-TiO_2_/m-TiO_2_/Perovskite/HTM; Figure S3: The pathway of current in the single cell and three interconnected cells; Figure S4: The IV curves of single cell and interconnected cell; Figure S5: The IV curves of single cell and interconnected cell; Figure S6: X-ray diffraction (XRD) pattern of perovskite films made by PbI_2_/DMF and Pb(NO_3_)_2_/water two-step dipping procedure; Figure S7: The photograph of Pb(NO_3_)_2_ films made by (a) 1.5 M (b) 1.6 M (c) 1.7 M and (d) 1.8 M Pb(NO_3_)_2_/water two-step dipping procedure, and corresponding SEM images of defect on 1.8 M Pb(NO_3_)_2_ films; Table S1: Cost analysis of materials for PbI_2_/DMF and Pb(NO_3_)_2_/water two-step dipping procedure under academic research; Table S2: Cost analysis of materials for PbI_2_/DMF and Pb(NO_3_)_2_/water two-step dipping procedure under industrial production; Table S3: Cost analysis of materials for PbI_2_/DMF and Pb(NO_3_)_2_/water two-step dipping procedure under industrial production.
