# Supplementary material for: Toward Clean and Economic Production of Highly Efficient Perovskite Solar Module Using a Cost-Effective and Low Toxic Aqueous Lead-Nitrate Precursor
Source: Nanomaterials (Basel). 2022 Oct 27;12(21):3783. doi: 10.3390/nano12213783 (PMC9655964; doi:10.3390/nano12213783)
Supplement: Supplementary file 1 [file nanomaterials-12-03783-s001.zip › nanomaterials-1956880-supplementary.pdf]

Supporting Information

Toward Clean and Economic Production of Highly Efficient Perovskite Solar Module Using a Cost-Effective and Low Toxic Aqueous Lead-Nitrate Precursor

Yi-Chen Teng [1], Tzu-Sen Su [1,*], Shiang Lan [2], Ahmed Fouad Musa [1] and Tzu-Chien Wei [1,*]

[1] Department of Chemical Engineering, National Tsing Hua University, 101, Section 2, Kuang Fu Road, Hsinchu 30013, Taiwan
[2] Taiwan Perovskite Technology Co., Ltd., 2F, No. 33, Section 1, Jiafeng 11th Road, Zhubei City 302052, Taiwan

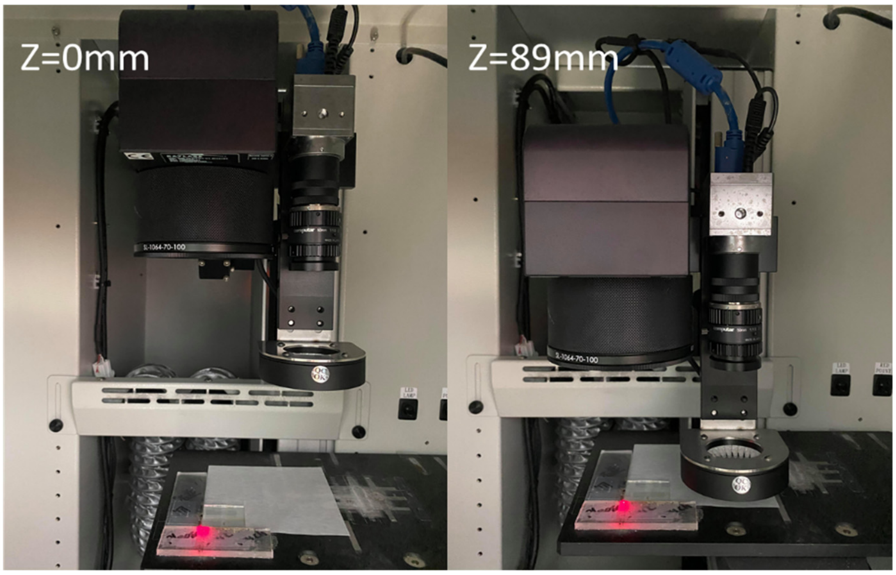

Figure S1. The control of focal length at Z axis.

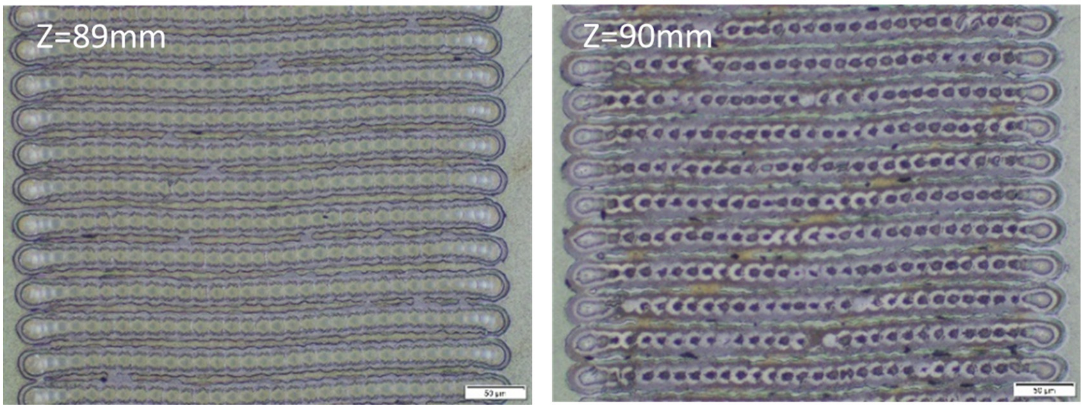

Figure S2. The optical microscopy images of 89 and 90mm focal length of P2 scribing on FTO/c-TiO_2/m-TiO_2/Perovskite/HTM

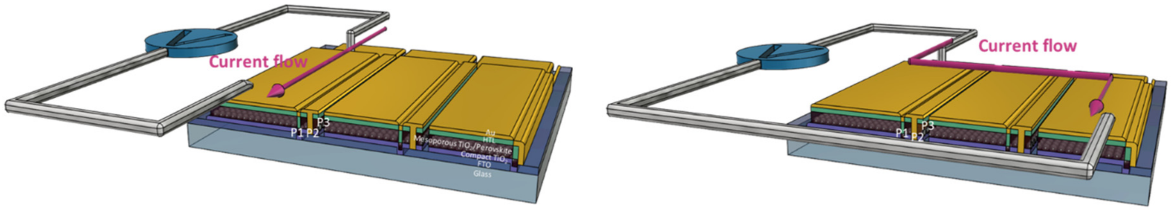

Figure S3. The pathway of current in the single cell and three interconnected cells.

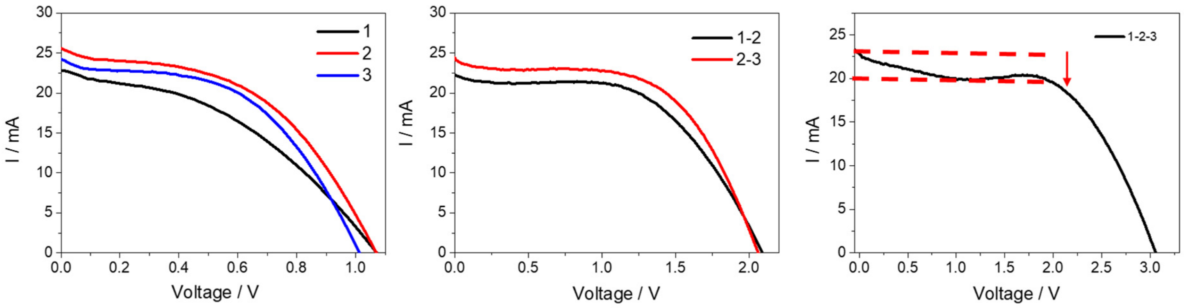

Figure S4. The IV curves of single cell and interconnected cell.

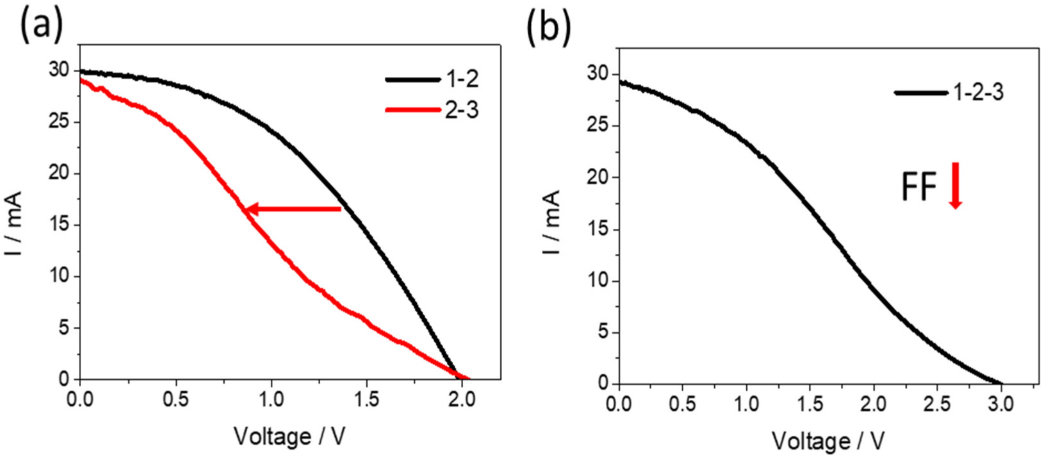

Figure S5. The IV curves of single cell and interconnected cell.

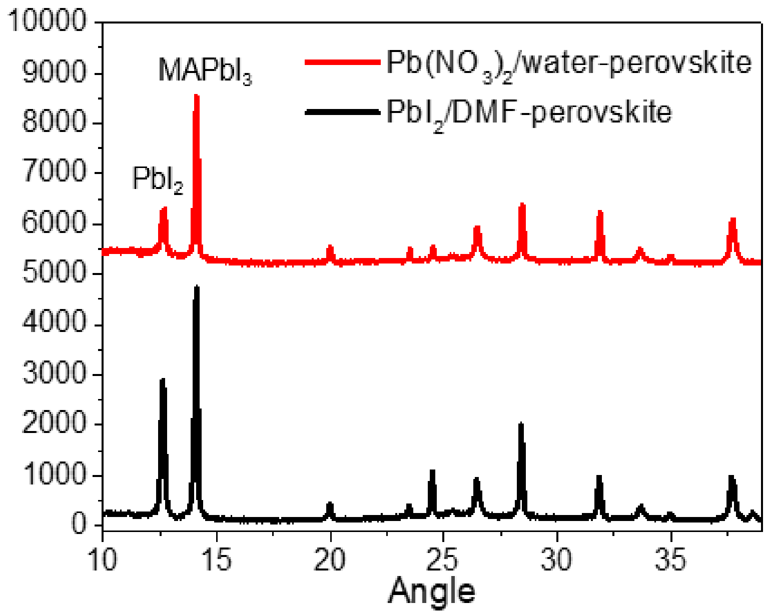

Figure S6. X-ray diffraction (XRD) pattern of perovskite films made by PbI_2/DMF and $Pb(NO_3)_2$/water two-step dipping procedure.

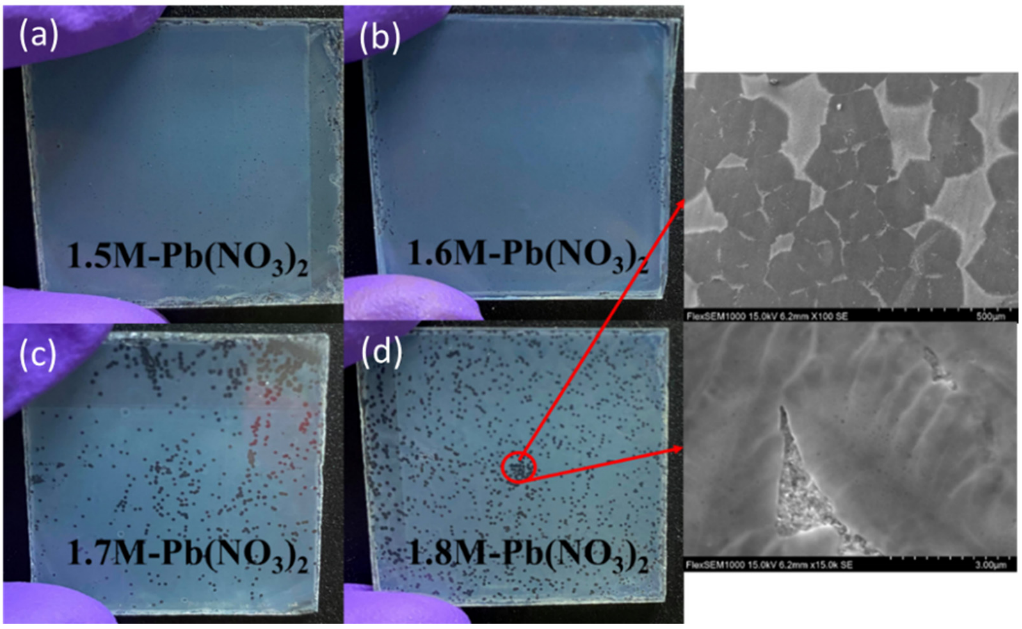

Figure S7. The photograph of $Pb(NO_3)_2$ films made by (a) 1.5M (b)1.6M (c) 1.7M and (d) 1.8M $Pb(NO_3)_2$/water two-step dipping procedure, and corresponding SEM images of defect on 1.8M $Pb(NO_3)_2$ films.

Table S1. Cost analysis of materials for PbI_2/DMF and $Pb(NO_3)_2$/water two-step dipping procedure under academic research.

		PbI_2/DMF protocol			$Pb(NO_3)_2$/water protocol		
Chemical	Purity	Price	Amount	Cost	Price	Amount	Cost
PbI_2	99.999%	25.8 USD/g	0.17 g	4.46 $	-	-	-
$Pb(NO_3)_2$	99.999%	-	-	-	5.99 $/g	0.13 g	0.64$
DMF	≥99.9%	0.15 USD/mL	0.23mL	0.03$	-	-	-
DMSO	≥99.9%	0.32 USD/mL	0.03mL	0.01$	-	-	-
DI water	-	-	-	-	~0 $/mL	0.25mL	~0 $
Efficiency of PSM		PCE=10.75 %			PCE=13.10 %		
Total cost per W		**99.74 $/W**			**14.42 $/W**		

Note: (a) Active area = 4.2 cm^2; (b) The price of material and solvent are taken from Sigma-Aldrich (USA) website. (c) The high quality and amount of materials are selected by the academic research of requirement.

Table S2. Cost analysis of materials for PbI_2/DMF and $Pb(NO_3)_2$/water two-step dipping procedure under industrial production.

		PbI_2/DMF procedure			$Pb(NO_3)_2$/water procedure		
Materials	Purity	Price	Amount	Cost	Price	Amount	Cost
PbI_2	99%	1.04$/g	0.17 g	0.18 $	-	-	-
$Pb(NO_3)_2$	99%	-	-	-	0.44 $/g	0.13 g	0.06$
DMF	99.8%	0.06$/mL	0.23mL	0.01$	-	-	-
DMSO	99.9%	0.18$/mL	0.03mL	0.01$	-	-	-
DI water	-	-	-	-	~0 $/mL	0.25mL	~0 $
Efficiency of PSM		PCE=15.00%			PCE=15.00%		
Total cost per W		**3.14 $/W**			**0.92 $/W**		

Note: (a) Active area = 4.2 cm^2; (b) The price of material and solvent are taken from Sigma-Aldrich (USA) website. (c) The quality and amount of materials are selected by the industrial production of requirement.

Table S3. Cost analysis of materials for PbI_2/DMF and $Pb(NO_3)_2$/water two-step dipping

procedure under industrial production.

		PbI_2/DMF procedure			$Pb(NO_3)_2$/water procedure		
Materials	Purity	Price	Amount	Cost	Price	Amount	Cost
PbI_2	99%	1.04$/g	0.17 g	0.18 $	-	-	-
$Pb(NO_3)_2$	99%	-	-	-	0.44 $/g	0.13 g	0.06$
DMF	99.8%	0.06$/mL	0.23mL	0.01$	-	-	-
DMSO	99.9%	0.18$/mL	0.03mL	0.01$	-	-	-
DI water	-	-	-	-	~0 $/mL	0.25mL	~0 $
Efficiency of PSM		PCE=20.00%			PCE=15.00%		
Total cost per W		**2.36 $/W**			**0.92 $/W**		

Note: (a) Active area = 4.2 cm^2; (b) The price of material and solvent are taken from Sigma-Aldrich (USA) website. (c) The quality and amount of materials are selected by the industrial production of requirement.
